# The correlation between pain and function in cancer survivors

**DOI:** 10.1007/s00520-026-10988-1

**Published:** 2026-07-09

**Authors:** Sean R. Smith, Jared Gershowitz, Nicholas Stoyles, Blair Richards

**Affiliations:** 1 Department of Physical Medicine and Rehabilitation, University of Michigan, 325 E Eisenhower Pkwy, Ste 100, Ann Arbor, MI USA; 2 CentraCare Health, St. Cloud, MN USA; 3 Michigan Institute for Clinical and Health Research, University of Michigan, Ann Arbor, MI USA

**Keywords:** Cancer pain, Cancer rehabilitation, Function, Physical function and cancer, Patient reported outcome measures, Rehabilitation outcomes

## Abstract

**Background:**

Function is critically important to cancer survivors, who are at risk of losing independence and mobility. But relatively, little is known about how pain, a widely prevalent symptom, contributes to functional decline. Understanding this may lead to earlier intervention and improved clinical outcomes.

**Methods:**

Four hundred eighty-eight participants across five cancer centers with rehabilitation clinics completed the PROMIS Cancer Function Brief 3D profile assessment and the numeric pain rating scale (NRS) during ambulatory clinic visits. Key clinical and demographic factors, including age, cancer diagnosis and status, cancer treatment history, age, and the presence of potentially function-limiting non-cancer factors, were recorded. Pearson correlation coefficients were calculated to determine the relationship between NRS scores and the PROMIS domains, and a multivariable analysis followed by a post-hoc least square mean analysis was conducted to explore relationships between clinical and demographic factors and reduced function and higher pain.

**Results:**

Patient-reported pain was moderately correlated with patient-reported physical function (*r* = 0.38), fatigue (*r* = −0.44), and social participation (*r* = 0.44). The presence of a non-cancer musculoskeletal impairment (*p* < 0.001) and of metastatic disease (*p* = 0.002) was significantly associated with higher pain levels. Age, the presence of active cancer (compared to no evidence of disease), the use of an assistive device for ambulation, the presence of chemotherapy-induced peripheral neuropathy, and the presence of a non-cancer psychiatric co-morbidity were not significantly associated.

**Conclusions:**

Cancer survivors with higher levels of pain are more likely to report functional decline. Non-cancer musculoskeletal factors are strong contributors to this pain, as is the presence of metastatic disease.

## Introduction

As advances in cancer treatment prolong survival, a growing number of cancer survivors—any patient diagnosed with cancer—experience or are at high risk of developing a loss of function due to symptoms resulting from cancer and cancer treatment. These symptoms negatively affect components of function, including physical mobility, independence with activities of daily living, participation in social and vocational roles, and health-related quality of life (HRQoL), while increasing caregiver strain and healthcare utilization [1]. This is true for both patients actively receiving treatment and survivors who have completed treatment. Accordingly, patient preferences for cancer treatment shift towards maintaining function and independence [2–5]. This decline in function is also associated with decreased survival in people with a history of cancer, necessitating an urgent examination of the risk factors for and correlating symptoms contributing to reduced mobility. Additionally, reduced function may hinder access to optimal cancer treatment, leading to treatment dose reductions, treatment delays, and exclusion from clinical trials [6, 7]. Given these profound implications, a deeper understanding of functional decline, which may inform mitigation and early intervention strategies, is essential.

Multiple symptoms related to cancer may contribute to impaired function in survivors, often manifesting as symptom clusters. While reduced physical activity has been associated with reduced HRQoL and increased fatigue, importantly, physical activity (how much a person moves) is not the same as function (how well and independently a person conducts activities) [8, 9]. Function, on the other hand, has not been extensively studied as it relates to symptoms. Within symptom clusters, pain is a common and debilitating symptom among cancer survivors, yet its contribution to functional decline is not well understood [10]. Cancer and its treatments, including surgery, chemotherapy, radiation, endocrine therapy, stem cell and engineered T-cell therapy, and immune checkpoint inhibitor therapy, can induce both acute and chronic pain through inflammation, direct neurologic damage, musculoskeletal injury, and more [11]. Considering its high prevalence and significant burden on the healthcare system, clarifying the relationship between cancer-related pain and movement limitations is essential for identifying key contributors to functional decline and developing targeted interventions to preserve function and improve patient outcomes.

This study is aimed at clarifying the role of pain in functional decline among cancer survivors. The primary objective is to examine the relationship between patient-reported function and pain in individuals with a history of cancer. A secondary objective is to identify clinical factors associated with pain and/or functional decline, providing insight into potential avenues for further exploration and intervention. Investigating these associations will inform subsequent research and clinical strategies aimed at exploring causality and optimizing function and enhancing HRQoL in cancer survivors.

## Methods and participants

A cross-sectional convenience sample of patients age 18 + with a history of cancer being treated in outpatient cancer rehabilitation medicine clinics across five performance sites was obtained. Patients with a history of any cancer, at any stage in treatment, were potentially eligible. Participants were excluded if they had a cognitive or communication deficit impairing their ability to read and complete the outcome measures, or if they did not speak and read English. Eligible patients completed written consent forms.

Enrolled participants completed the PROMIS Cancer Function Brief 3D profile, a validated patient-reported outcome measure that assesses physical function, as well as fatigue and social participation over a patient’s previous 7 days, that has been shown to be responsive to changes in function when a patient’s pain changes [12, 13]. Patients also completed the numeric pain rating scale (NRS), asking “How would you rate your pain on average” and rating their pain on a scale from zero (no pain) to 10 (worst imaginable pain). The NRS was selected due to its ease of use, short completion time to limit subject response burden, and validation studies finding good sensitivity, responsiveness, and reliability, and ability to be statistically analyzed in comparison to other outcome measures [14–17]. Demographic and clinical data were recorded by the treating physicians, including age, body mass index, cancer type, cancer treatment history (surgery, radiation, chemotherapy, endocrine therapy, and immune checkpoint inhibitor therapy), and cancer status (active disease or no evidence of disease, and presence of bone, brain, and/or other distant metastases). Additionally, other factors potentially contributing to pain and function were recorded, including the presence of non-cancer musculoskeletal and psychiatric comorbidities, and the use of an assistive device for ambulation (wheelchair, cane/walker, or none) and the presence of chemotherapy-induced peripheral neuropathy (CIPN) as determined by the treating physician.

Prior to study initiation, institutional review board approval was obtained at each performance site. The coordinating site was responsible for data management.

### Statistical analyses

Pearson’s correlation coefficients were calculated to determine the relationship between NRS scores and the three domains of the PROMIS Cancer Function Brief 3D Profile (physical function, fatigue, and social participation). First, univariable comparisons between pain levels and clinical factors associated with lower function were performed using the ANOVA (categorical variables) and linear regression (continuous variables). Clinical factors with a *p* < 0.10 from the univariable analysis were selected for inclusion into a ANCOVA multivariable analysis.

Post hoc analyses of least squares means (95% confidence intervals [CI]) were conducted if the overall effect of a clinical variable on pain rating was statistically significant to evaluate group differences. For 2-tailed *p* values, a value of < 0.05 was considered statistically significant. Statistical Analysis Software (SAS) 9.4 (Cary, NC, USA) was used for the analyses.

## Results

A total of 488 participants were enrolled in the study. The mean age was 57.5, and descriptive statistics, including the number of patients with a given covariate potentially limiting function, are in Table 1. The mean *T*-score of each domain was 40.2 (physical function), 55.6 (fatigue), and 44.8 (social participation), where 50.0 is the population mean and higher T-scores indicate that the patient experiences more of a given domain. Pearson correlation coefficients found moderate correlations between patient-reported pain and physical function (*r* = −0.38), fatigue (*r* = −0.44), and social participation (*r* = −0.44) [18] (Table 2) The univariable analysis subsequently found potential contributions from multiple factors that met statistical significance (*p* < 0.05), including the presence of metastatic disease (*p* = 0.014, parameter estimate: 0.71 [95% CI: 0.14 to 1.27]) and the presence of non-cancer musculoskeletal (severe impairment, *p* < 0.0001, parameter estimate: 2.80 [95% CI: 1.82 to 3.79], moderate impairment 1.36 [95% CI: 0.89 to 1.84]) or psychiatric co-morbidities (severe impairment, *p* = 0.020, parameter estimate: 1.52 [95% CI: −0.04 to 3.07], moderate impairment 0.82 [95% CI: 0.07 to 1.58]).

**Table 1:** Patient characteristics

Characteristic		Cohort (*n* = 488)
Age (yrs)	Mean (SD)	57.5 (13.8)
	Median (IQR)	59.0 (49.0 to 68.0)
	Range	21.0 to 95.0
	N	488
Active cancer	No	299 (61)
	Yes	189 (39)
Total # of cancers	1	454 (93)
	2	28 (6)
	3	6 (1)
Cancer type	Breast	209 (43)
	Head/neck, non-thyroid	33 (7)
	Thyroid	4 (1)
	Prostate	18 (4)
	Brain	27 (6)
	Sarcoma	28 (6)
	Melanoma	6 (1)
	Colorectal	15 (3)
	Bladder/ureteral	3 (1)
	Renal	6 (1)
	Lung	9 (2)
	Gynecologic	23 (5)
	Multiple myeloma	21 (4)
	Leukemia, no alloHSCT	4 (1)
	Lymphoma, no alloHSCT	12 (2)
	History of alloHSCT	14 (3)
	Other	22 (5)
	Multiple cancers	34 (7)
Metastatic disease	No	396 (81)
	Yes	92 (19)
Brain metastases	No	474 (97)
	Yes	14 (3)
Bone metastases	No	429 (88)
	Yes	59 (12)
CIPN	No	387 (79)
	Yes	101 (21)
Non-cancer psychiatric impairment	None/minimal	432 (89)
	Moderate	46 (9)
	Severe	10 (2)
Non-cancer musculoskeletal impairment	None/minimal	324 (66)
	Moderate	140 (29)
	Severe	24 (5)
Ambulation status	Wheelchair	22 (5)
	Modified independent	74 (15)
	Independent	392 (80)
Chemotherapy	Never	124 (25)
	Previous	257 (53)
	Active	107 (22)
Radiation therapy	Never	171 (35)
	Previous	304 (62)
	Active	13 (3)
Surgery for cancer	Never	100 (20)
	4 weeks or more ago	376 (77)
	Within 4 weeks	12 (2)

Interpretation: values are mean (SD) or *n* (%)

*CIPN* chemotherapy-induced peripheral neuropathy, *alloHSCT* allogeneic hematopoietic stem cell transplantation

**Table 2:** Pain and function correlations

Variable	Pearson’s correlation (*r*)	*p* value
Physical function scale (*n* = 488)	−0.38	< 0.001
Fatigue scale (*n* = 486)	0.44	< 0.001
Social participation scale (*n* = 480)	−0.44	< 0.001

Factors with *p* < 0.10, and therefore included in the multivariable analysis, included having CIPN (*p* = 0.091, parameter estimate: 0.47 [95% CI: −0.08 to 1.02]) and the use of a wheelchair or assistive device for ambulation (*p* = 0.064, parameter estimate: 1.28 [95% CI: 0.10 to 2.47]).

The multivariable analyses that assessed all predictors a priori, and all with *p* < 0.10 on the univariable analysis both found a significant association between reported pain levels and the presence of metastatic disease (*p* = 0.0020, parameter estimate: 0.86 [95% CI: 0.32 to 1.40]) and non-cancer musculoskeletal impairment (moderate 1.26 [95% CI: 0.79 to 1.74] and severe 2.79 [95% CI: 1.78 to 3.80]) (Table 3).

**Table 3:** Univariable and multivariable results

**Variable**	**Univariable analysis (*n* = 488)**		**Multivariable analysis (*n* = 488)**	
	**Estimates (CI)**	***p* value**	**Estimates (CI)**	***p* value**
Intercept			3.15 (2.84, 3.45)	<.001
Metastatic disease (Ref: no)	0.71 (0.14, 1.27)	0.014	0.86 (0.32, 1.40)	0.002*
CIPN (Ref: no)	0.47 (−0.08, 1.02)	0.091	0.21 (−0.32, 0.73)	0.438
Psychiatric impairment (Ref: none/minimal)	Overall effect	0.020	Overall effect	0.083
Moderate	1.52 (−0.04, 3.07)	0.057	0.62 (−0.10, 1.34)	0.094
Severe	0.82 (0.07, 1.58)	0.033	1.19 (−0.30, 2.67)	0.116
Musculoskeletal impairment (Ref: none/minimal)	Overall effect	<.001	Overall effect	<.001*
Moderate	2.80 (1.82, 3.79)	<.001	1.26 (0.79, 1.74)	<.001*
Severe	1.36 (0.89, 1.84)	<.001	2.79 (1.78, 3.80)	<.001*
Ambulation status (Ref: independent)	Overall effect	0.063	Overall effect	0.186
Modified independent	0.58 (−0.04, 1.20)	0.065	0.01 (−0.60, 0.62)	0.974
Wheelchair	−0.70 (−1.77, 0.37)	0.199	−0.94 (−1.96, 0.07)	0.069
Cancer status, active (Ref: non-active)	0.26 (−0.20, 0.72)	0.262		
Age	0.01 (−0.01, 0.02)	0.473		

Interpretation: for categorical predictors (e.g., CIPN), estimates represent the unit change in the pain score compared to the reference group. For continuous predictors (e.g., age), estimates represent the unit change in pain score per one unit increase in the predictor. *p* values less than 0.05 were deemed significant

*CIPN* chemotherapy induced peripheral neuropathy

## Discussion

This study found that patient-reported pain and physical function in adults with a history of cancer are moderately negatively correlated. Additionally, higher levels of pain were moderately correlated with higher fatigue and lower social participation. This, coupled with previous data showing that the patient-reported function changes as pain changes, suggests that, when present, pain is associated and may be a significant contributor to reduced function [13]. The implications for this are significant—patients suffering from reduced function during or after cancer treatment may need pain optimized in order to sufficiently restore, or minimize continued loss of function. This is critically important, as function is strongly associated with HRQoL in people with a history of cancer, and many cancer treatments require relatively robust function to tolerate toxic treatment regimens.

These results are also notable for identifying which function-reducing factors are most strongly correlated with pain. Unsurprisingly, the presence of metastatic disease—and not simply having active disease versus no evidence of disease—was associated with higher levels of pain. This is consistent with many other studies and supports the validity of this study’s overall findings [19–21]. Perhaps most striking, however, was that of all the potential covariates considered, the presence of non-cancer musculoskeletal conditions that limit function was most strongly associated with pain during or after cancer treatment. This suggests that patients with disability or reduced function with to musculoskeletal impairments (e.g., arthritis and back pain) may need closer monitoring of their pain and function, and a lower threshold to a specialty referral, such as physical medicine and rehabilitation or pain medicine, to optimize function. Also, and perhaps surprisingly, higher levels of pain were not strongly associated with function-limiting psychiatric conditions (e.g., fibromyalgia or severe depression), age, the presence of chemotherapy-induced peripheral neuropathy, and more.

There are, of course, limitations to this work. First, while valid and clinically useful, the NRS is limited in scope. It does not differentiate between types of pain (e.g., neuropathic, somatic, and nociplastic). Nor does it specify how pain interferes with daily life, though non-cancer studies have found that it links to pain interference in patients with spinal cord injuries [22]. It also does not ask specific questions about pain, such as would be seen in other pain assessment tools, preventing the clinician from viewing item-level knowledge to glean more specific information about a patient’s pain. For example, since it asks for an average level of pain, it does not provide detailed information regarding whether a patient is experiencing more or less pain when answering the question, potentially skewing the results. Finally, the question about pain asked a patient for an average level of pain, but the PROMIS items asked patients about their function over the past week. It is possible that a patient had higher or lower function over a 7-day period that was independent of their pain on the day of completing the NRS.

An additional limitation is that the study sample is for patients who were referred to physical medicine and rehabilitation physicians specializing in cancer rehabilitation—so the results suggest that amongst patients with reduced function, musculoskeletal impairments and metastatic disease are strongly correlated with pain. However, the results may differ if taken from a general oncology population.

The variety of cancers, as well as preponderance of breast cancer, in the sample can also be viewed as a limitation. While breast cancer is, by a wide margin, the most common cancer seen in cancer rehabilitation medicine clinics [23], it represents over 40% of the cohort in this study. While this represents a “real world” proportion of patients seen in these clinics, it limits the generalizability of the findings in this study for other disease types. Furthermore, issues like lymphedema and post-mastectomy pain, for which many breast cancer patients are seen in rehabilitation clinics, may have less pain and/or higher function than other patients in these clinics and may explain why the presence of metastatic disease—where those issues may be less of a factor in a patient’s rehabilitation needs—was so strongly correlated with higher pain and reduced function.

To our knowledge, this is perhaps the first study to evaluate correlations between pain and function across multiple domains in people with a history of cancer. Future studies may explore causality between pain and reduced function, or look at the types of pain that lower function in people with a history of cancer (e.g., neuropathic, somatic, or nociplastic). Additionally, clinical trials may measure function as a clinical endpoint when testing interventions to lower pain, and use pain as an endpoint when testing interventions to improve function. Finally, evaluating pain and function among all cancer patients, not just those referred for rehabilitation, would provide useful insight into pain and functional trajectories in this population.

## Data Availability

Data from this project are available upon request.
